# Cardiac Extracellular Vesicles in Normal and Infarcted Heart

**DOI:** 10.3390/ijms17010063

**Published:** 2016-01-05

**Authors:** Dimitry A. Chistiakov, Alexander N. Orekhov, Yuri V. Bobryshev

**Affiliations:** 1 Department of Molecular Genetic Diagnostics and Cell Biology, Division of Laboratory Medicine, Institute of Pediatrics, Research Center for Children’s Health, 119991 Moscow, Russia; dimitry.chistiakov@lycos.com; 2 Laboratory of Angiopathology, Institute of General Pathology and Pathophysiology, Russian Academy of Medical Sciences, 125315 Moscow, Russia; a.h.opexob@gmail.com; 3 Institute for Atherosclerosis Research, Skolkovo Innovative Center, 143025 Moscow, Russia; 4 Department of Biophysics, Biological Faculty, Moscow State University, 119991 Moscow, Russia; 5 Faculty of Medicine, School of Medical Sciences, University of New South Wales, Sydney, NSW 2052, Australia; 6 School of Medicine, University of Western Sydney, Campbelltown, NSW 2560, Australia

**Keywords:** extracellular vesicles, exosomes, microparticles, cardiomyocyte, acute myocardial infarction, cardiac repair

## Abstract

Heart is a complex assembly of many cell types constituting myocardium, endocardium and epicardium that intensively communicate to each other in order to maintain the proper cardiac function. There are many types of intercellular intracardiac signals, with a prominent role of extracellular vesicles (EVs), such as exosomes and microvesicles, for long-distant delivering of complex messages. Cardiomyocytes release EVs, whose content could significantly vary depending on the stimulus. In stress, such as hypoxia, inflammation or injury, cardiomyocytes increase secretion of EVs. In hypoxic conditions, cardiac EVs are enriched with angiogenic and prosurvival factors. In acute myocardial infarction (AMI), damaged cardiac muscle cells produce EVs with increased content of angiogenic, anti-apoptotic, mitogenic and growth factors in order to induce repair and healing of the infarcted myocardium. Exosomal microRNAs play a central role in cardiac regeneration. In AMI, circulating cardiac EVs abundantly contain cardiac-specific miRNAs that serve as indicators of cardiac damage and have a big diagnostic potential as AMI biomarkers. Cardioprotective and regenerative properties of exosomes derived from cardiac and non-cardiac stem/progenitor cells are very helpful to be used in cell-free cardiotherapy and regeneration of post-infarct myocardium.

## 1. Introduction

Communication between cells is essential, because this is the way by which cells contact each other and transfer pieces of information or signals. Indeed, an acceptor cell could change its behaviour or function in response to a signal. Cell-to-cell communication is necessary to maintain tissue/organ integrity/homeostasis and induce adaptive changes in response to exogenous stimuli. Many types of cell–cell communicative mechanisms are observed, including direct cell–cell connections, electrical stimuli, extracellular matrix interactions, release of various chemical substances and distant contacts. In a case of long-range contacts, cells release membrane-surrounded structures called extracellular vesicles (EVs). EVs carry a variety of molecules, including low molecular compounds, lipids, proteins and genetic material, such as DNA, mRNA and non-coding RNA. Indeed, EVs could serve as vehicles responsible for horizontal gene transfer between cells [1]. In EVs, intravesicular cargo could greatly vary depending on the cell type and microenvironment. EVs released by the same cell type could contain different repertoires of molecules in normal and pathogenic conditions. In addition, EVs vary by size and have different mechanisms of biogenesis and release from the cell.

## 2. Types of Extracellular Vesicles

Although the current EV nomenclature is not perfect, these particles could be divided by cell origin and size into exosomes, microvesicles (MVs) and apoptotic bodies (ABs) [2].

### 2.1. Exosomes

Among EVs, exosomes are the smallest particles that range in size from 40 to 100 nm and originate from cellular endosomes [3]. Exosomes are generated in endosomal vesicles called multivesicular bodies (MVBs). They result from the inside budding of the plasma membrane into early endosomes. Indeed, early endosomes have an inverted plasma membrane. During exosome formation, the future exosomal cargo contacts the outer endosomal membrane, which leads to the invagination of the membrane into the lumen and the gripping vesicle into the endosomal lumen. Therefore, due to the double membrane invagination during formation, exosomes have the same membrane orientation as the plasma membrane has.

Independently of the cell type, exosome membrane contains similar integral proteins, such as tetraspanins CD63, CD81 and CD82. Tetraspanins are present in released exosomes and accumulate in plasma membrane microdomains and endosomes. In the membrane, tetraspanin clusters form special tetraspanin-enriched microdomains (TEMs) that are involved in exosome biogenesis, exosome internalization, the selection of exosome cargo (*i.e.*, protein sorting) and antigen presentation (in immune exosomes) [4]. Endosomes are enriched by chaperones, such as heat shock protein (HSP)-27, HSP-60, HSP-70 and HSP-90. Chaperones participate in protein folding/unfolding and assembly/disassembly [5]. MVBs contain high amounts of the endosomal sorting complex required for transport (ESCRT), a protein machinery that is implicated in cargo recognition and loading, endosome membrane remodelling and endosome trafficking to lysosomes or to the plasma membrane to degrade or release endosomal content [6]. The cell endosomal system regulates both the uptake and processing of extracellular molecules and exchanging protein and lipids with the Golgi network in order to maintain cell homeostasis [7].

MVBs were found to be located close to processing bodies (P-bodies; cytoplasmic compartments consisting of enzymes involved in RNA turnover) containing trinucleotide repeat-containing gene 6A protein (TNRC6A; also known as GW182) and Argonaute-2 (AGO2), *i.e.*, functional components of the miRNA-loaded RNA-induced silencing complex (RISC) [8]. Gibbings *et al.* [8] found exosome-like vesicles enriched with TNRC6A that could suggest a potential role of AGO2 and TNRC6A in microRNA (miRNA) sorting before exosomal packaging. This could be supported by observations of the involvement of AGO2 and TNRC6A in the loading of Epstein-Barr virus-encoded miRNAs to exosomes that then are transported to recipient cells [9]. Exosomes are constitutively released by the fusion of MVBs to the plasma membrane. This mechanism is controlled by Rab GTPases, such as Rab27a and Rab27b [10]. Knockdown of Rab27a led to increased MVB size, while Rab27a silencing resulted in the redistribution of MVBs to the perinuclear region [11]. Indeed, these observations suggest that Rab27a and Rab27b regulate different steps of exosome secretion. Recently, Mazzeo *et al.* [12] showed the involvement of members of the protein kinase D (PKD) family in MVB maturation and exosome release. PKD1/2 activity and subcellular localization are regulated by diacylglycerol kinase α (DGKα). PKD1/2 acts as a mediator of the DGKα effect on MVB movement to the plasma membrane [12]. Inducible secretion of exosomes could be initiated by various stimuli and depends on the cell type.

### 2.2. Microvesicles

MVs (also called ectosomes and microparticles) are larger than exosomes (size range 100 to 1000 nm). Except for the size, microvesicles differ from exosomes by the mechanisms of release and biogenesis. MVs are shed through outward budding and fission of membrane vesicles from the plasma membrane [13]. In many ways, the fission resembles the abscission step in cytokinesis [14]. MV shedding also shares similarities with the mechanism of virus budding. For example, retroviral Gag proteins that are necessary for virion assembly cluster at the plasma membrane and induce its outward protrusion. The viral bud in turn releases when the bud neck is pinched behind the virion [15].

MVs are shed by various cells, especially by platelets, endothelial cells and erythrocytes. Compared to exosomes that are more constitutively formed and released, MVs appear to be produced in response to stimuli [16]. MVs were defined by their capacity to bind to annexin V, an adhesion molecule that specifically interacts with phosphatidylserine [17]. However, some MVs failed to bind to annexin V or lactadherin, but engage duramycin, a phosphatidylethanolamine-specific peptide [18], suggesting the enrichment of the membrane of some microvesicular populations with this phospholipid.

Like exosomes, MVs carry a variety of molecules. Since MVs are inducible, their composition could be frequently enriched with bioactive molecules whose production is specifically induced in response to a certain stimulus. For example, in prothrombotic conditions, platelets release large-sized MVs enriched with factors that stimulate the endothelial barrier function. After thrombus formation, platelet-derived MVs predominantly contain factors that inhibit thrombogenesis [19].

### 2.3. Apoptotic Bodies

ABs are the largest EVs, whose size varies between 1 and 5 μM. These particles are released by apoptotic cells as blebs. AP blebbing is regulated by activity Rho-associated kinase 1 (ROCK1) [20]. Caspase-3 was shown to constitutively activate ROCK1 that, in turn, phosphorylates myosin light chain (MLC) and induces membrane blebbing [21]. ABs can contain whole organelles and nuclear fragments, such as nucleosomal histones and fragmented DNA [22]. Phosphorylation of MLC and the activity of MLC ATPase leads to the actin-myosin cytoskeletal contraction that disrupts nuclear integrity. This, in turn, causes chromosomal DNA fragmentation followed by reallocation of DNA fragments to blebs and ABs [23]. AP release serves as a signal stimulating phagocytosis of apoptotic cells before induction of secondary necrosis [24]. APs are enriched with various damage-associated molecular pattern proteins (DAMPs) that can induce inflammation [25].

## 3. Intracardiac Communication

Many cell types are involved in proper heart function, including cardiomyocytes, myofibroblasts, endothelial cells (ECs), vascular smooth muscle cells, neuronal cells (sympathetic and parasympathetic), immune cells and cardiac-derived stem cells [26,27]. All of this cell assembly should act in a strictly coordinated fashion to support cardiac automaticity and rhythm, regular blood supply and reliable changes in heart function in response to exogenous stimuli. Indeed, well-regulated and well-balanced intracardiac communication is needed to control heart integrity and homeostasis.

A complex intrinsic signaling network exists between cardiac muscle cells and between cardiomyocytes and other heart cells to respond to stimuli. To work properly, the heart muscle requires a large amount of oxygen that exceeds the oxygen supply needed for the rest of skeletal muscle. Indeed, the cardiac artery vasomotor function should be efficiently performed and mediated by a coordinated release of various vasoconstrictors and vasodilators by ECs in response to metabolic, neuronal and hormonal signals [28].

During cardiogenesis, paracrine signaling, including Wnt-dependent and fibroblast growth factor (FGF)-dependent pathways, plays a key role in heart development [29]. However, along with local secretion of free growth factors and other signaling molecules, EV-mediated intercellular contacts in the myocardium and between the myocardium, endocardium and epicardium also play a role, especially at long distances [27]. EV-dependent communication could be superior compared to the release of free soluble factors. First, EV cargo is well protected by the membrane. Second, vesicle-mediated transport might be better organized, because EVs could be specifically delivered to target cells. In this review, we will consider current knowledge on intracardiac communications mediated by EVs in normal and infarcted heart.

## 4. Cardiomyocytes Release Exosomes and Microvesicles Whose Cargo Could Be Influenced by Various Factors

Usually, cardiac muscle cells are not considered as typical secretory cells. However, they can produce exosomes and MVs, especially in an inducible manner. It should be mentioned that data currently available for cardiac exosomes were mainly produced *in vitro*, *i.e.*, in cell culture experiments. Gupta and Knowton [30] first isolated cardiac-specific exosomes released by adult rat cardiac myocytes and containing HSP-60. The release of cardiac exosomes was enhanced by hypoxia. Extracellular HSP-60 was shown to contribute to cardiomyocyte apoptosis and inflammation through a Toll-like receptor (TLR)-mediated mechanism [31,32].

Cardiomyocyte EVs had a size ranging from 40 to 300 nm (*i.e.*, corresponding to the size of exosomes and MVs) and contained caveolin-3 and flotillin-1 on their surface [33]. Proteomic analysis of cardiomyocyte-derived exosomes revealed the enrichment with sarcomeric and mitochondrial proteins, including tropomyosin, myomesin, cardiac-type myosin-binding protein C (MYBPC3) and valosin-containing protein (VCP) [34]. Indeed, compared to EVs derived from other cells, cardiac EVs have a specific proteomic signature that underlines its origin from the myocardium. The release of cardiac EVs could be promoted by alcohol intake in a manner similar to that of oxidative stress, indicating the potential involvement of reactive oxygen species (ROS)-mediated signaling in the stimulation of EV secretion by cardiac muscle cells. Exposure to alcohol or oxidative stress leads to changes in the cardiac EV proteome [34].

Waldenström *et al.* [33] showed that EVs secreted by cardiomyocyte are able to transfer nucleic acids. These EVs had a size ranging from 40 to 300 nm (*i.e.*, corresponding to the size of exosomes and MVs) and contained caveolin-3 and flotillin-1 on their surface. Cardiac EVs contained over 1500 different mRNA transcripts and 340 distinct DNA sequences. Transfer of cardiac EVs to cultured fibroblasts resulted in qualitative transcriptome changes, suggesting that cardiac EVs carry biologically-active factors capable of influencing gene expression [33].

Growth factors, such as platelet-derived growth factor (PDGF) and transforming growth factor (TGF)-β, could induce quantitative and qualitative changes in RNA transcripts carried by exosomes released by HL-1 cells, a cell line derived from the AT-1 mouse atrial cardiomyocyte tumour lineage [35,36]. However, over 200 common transcripts remained present in exosomes from TGF-β- and PDGF-treated and non-treated myocytes. These transcripts are enriched with ribosomal, cytosolic and mitochondrial transcripts related to the energy supply. Indeed, these findings suggest that cardiac exosomes transfer a basic package of transcripts involved in energy generation. Cardiomyocytes are known to contain a large number of mitochondria due to the high energy demand. Another characteristic of the cardiac muscle cell is the intensive protein turnover, which could explain high ribosomal mRNA content in exosomes. In TGF-β-stimulated exosomes, enrichment with mRNAs related to TGF-β-dependent signaling was detected [36]. TGF-β-dependent transcripts involve those that encode regulators of cell proliferation and cell growth, such as activated T-cells 5 (NFAT5) and histone deacetylase 5 (HDAC5), thereby indicating that TGF-β induces the release of cardiac exosomes enriched with proliferative and hypertrophic factors [36].

In cardiomyocytes, hypoxia is a potent stimulator of exosome release. For example, cardiomyocytes exposed to moderate hypoxia for 2 h increase exosome secretion nearly by two-fold [30]. Yu *et al.* [37] showed that exosomes secreted by primary cultured cardiomyocytes under hypoxia have a high content of tumour necrosis factor (TNF)-α, a proinflammatory cytokine whose cardiomyocyte expression is induced by hypoxia inducible factor (HIF)-1α. Indeed, hypoxia could induce the release of cardiac EVs that contain proteins with opposite activities (for example, pro-apoptotic and anti-apoptotic factors). The severity and duration of hypoxic exposure is likely to modulate the content of cardiac EVs.

## 5. Cardiac Exosomes Are Involved in Crosstalk between Cardiomyocytes and Endothelial Cells

Communications between cardiac muscle cells and intracardiac ECs play a crucial role in the regulation of the function of cardiac blood vessels involved in supporting the myocardium with oxygen and nutrients. EVs were shown to be involved in cardiac myocyte-endothelial contacts. In cardiomyocytes from newborn piglets subjected to hypoxia, HIF-1α upregulation was shown to lead to the induction of Hsp20 [38]. Hsp20 has an angiogenic activity inducing the expression of vascular endothelial growth factor (VEGF) receptor-2 (VEGFR2) in ECs that stimulates cardiac angiogenesis in response to hypoxia.

Exosomes derived from cardiac progenitor cells (CPCs) were shown to stimulate EC migration that may be helpful for cardiac repair and neovascularization of infarcted heart tissue [39]. However, in diabetic conditions, cardiac exosomes could display anti-angiogenic properties on vascular ECs mediated by exosomal transfer of microRNA-320 (miR-320) [40]. MicroRNAs (miRNAs) belong to a class of small regulatory RNAs that negatively control mRNA expression at the post-transcriptional and the post-translational level. Diabetes-induced aberrant overexpression of miR-320 in cardiac myocytes and further delivery of this miRNA to myocardial ECs by cardiac exosomes results in downregulation of the endothelial production of Hsp20, insulin-like growth factor-1 (IGF-1) and transcription factor Ets2 [40,41]. This could explain cardiac vascular dysfunction in diabetes. The mechanism of diabetic-dependent activation of miR-320 in cardiac myocytes is unclear, but probably is not mediated by hyperglycemia, since high glucose inhibits miR-320 expression in human umbilical vein endothelial cells (HUVECs) [42].

## 6. Effects of Exosomes Released by Cardiac Endothelial Cells on Cardiomyocytes

Cardiac muscle cells and ECs reciprocally communicate with each other, including EV-mediated mechanisms. In cardiac ECs, hypoxic conditions stimulate the expression of miR-126 and miR-210 [43]. Both miRNAs possess proangiogenic properties, especially miR-210, which is specifically induced by hypoxia [44]. In addition, miR-126- and miR-210-enriched exosomes from hypoxia-exposed ECs were shown to increase the resistance of CPCs to hypoxic stress through stimulation of PI3K/Akt and other prosurvival pathways [44,45].

In cardiac ECs from women affected with peripartum cardiomyopathy (a life-threatening pregnancy-associated cardiac pathology), the cathepsin D-cleaved 16-kDa N-terminal prolactin fragment upregulates the expression of miR-146 [46]. The prolactin fragment is involved in the pathogenesis of peripartum cardiomyopathy exhibiting anti-angiogenic and proapoptotic effects on the myocardium [47]. miR-146 also possesses anti-angiogenic properties via inhibiting endothelial production of neuroblastoma RAS viral (v-ras) oncogene homolog (NRAS). ECs from diseased hearts release miR-146-enriched exosomes that are taken up by cardiomyocytes. In cardiomyocytes, exosomal transfer of miR-146 targets key signaling pathways mediated by Erb-b2 receptor tyrosine kinase 4 (ERBB4), Notch1 and interleukin-1 receptor-associated kinase 1 (IRAK1), which in turn cause cardiometabolic and contractile impairments [46]. This leads to the development of cardiac hypertrophy, while the density of myocardial microvessels is reduced [48]. Thus, heart overexpression of miR-146 plays a pathological role in peripartum cardiomyopathy.

## 7. Cardiac Fibroblast-Derived Exosomes Influence Cardiac Muscle Cells

As cardiac EVs are able to influence fibroblasts [33], cardiac fibroblasts in turn secrete exosomes that exhibit effects on cardiomyocytes. Recently, Bang *et al.* [49] observed an unusual enrichment of cardiac fibroblast-derived exosomes with miRNA passenger strands (such as miR-21*) that are normally eliminated in miRNA biogenesis. These miRNAs were suggested to act as paracrine signaling mediators in the heart [50]. Bang *et al.* [49] showed that miR-21* transported to cardiac muscle cells with fibroblast-derived exosomes downregulates sorbin and SH3 domain-containing protein 2 (SORBS2) and PDZ and LIM domain 5 (PDLIM5), inducing hypertrophy. Both miR-21* targets play an important role in organizing cardiac muscle structure and function, and their downregulation results in cardiac hypertrophy [51,52,53]. Inhibition of miR-21* in mice with angiotensin II-induced heart hypertrophy suppressed hypertrophic growth of cardiac muscle cells [49]. Indeed, fibroblast exosomes could be involved in the delivery of the profibrotic and prohypertophic miR-21* to cardiac muscle cells.

## 8. Acute Myocardial Infarction

Except for heart physiology, cardiac exosomes play a role in various cardiac pathologic conditions, including acute myocardial infarction (AMI). Briefly, we consider the pathogenesis of AMI. Coronary atherosclerosis accompanied with the formation of intraluminal thrombus accounts for 80% of the cases of acute myocardial infarction (AMI) [54]. Rupture of the coronary artery plaque induces aggregation of platelets followed by thrombus formation and propagation along the coronary artery. Thrombus build-up in the diseased coronary artery causes acute ischemia and local necrosis of the downstream myocardial zone. The necrotic zone expands outwards towards the epicardium. In some sites (usually at the edges of the infarct), the myocardium becomes stunned and could then recover when the blood supply is restored. The remaining functional cardiac muscle increases contraction, a phenomenon called hyperkinesis [54].

In the infarcted region, the irreversible damage of cardiac cells occurs over 12 h post-MI [55]. Between 4 and 12 h after the beginning of cell death, the infarcted heart muscle undergoes coagulation necrosis characterized by the destruction of damaged cells. After 18 h, neutrophils appear in the infarcted zone forming a granulation tissue at the edges of the infarct by Days 3–4. This tissue also contains fibroblasts, macrophages and neovessels. The granulation tissue slowly moves to the infarct centre, replacing the necrotic zone, which is digested by macrophages. The granulation tissue is progressively replaced by the scar that has no capillaries. After 2–3 months, infarct healing is usually completed [55].

However, electrical conduction of post-infarct cardiac tissue is slower than that of the normal myocardium. The difference in conduction velocity between infarcted and non-infarcted tissue causes re-entrant arrhythmias [56]. Re-entrant arrhythmias could in turn progress to more dangerous arrhythmias, such as ventricular tachycardia, and, in turn, increase the risk of sudden death.

Cardiomyocyte death caused by myocardial injury induces the repair mechanism associated with extensive cardiac tissue remodelling and fibrosis. Myofibroblasts that arise from cardiac fibroblasts and other cells (ECs and circulating cells) are the main contributor to cardiac remodelling [57]. During fibrosis, ECs undergo endothelial-to-mesenchymal transition (EMT) driven by TGF-β_1_ released by fibroblasts [58]. Myofibroblasts produce collagens that deposit in the infarcted myocardium, forming connective tissue, or scar. In addition, cardiomyocytes surviving in the wounded myocardium release a variety of molecules that serve as a warning signal of myocardial damage essential for the induction of cardiac repair.

## 9. Exosomal Cardiac-Specific MicroRNAs as a Diagnostic Marker of Acute Myocardial Infarction

In AMI, myocardial injury leads to rapid appearance of cardiomyocyte-specific biomolecules in the bloodstream, a phenomenon that is helpful for early diagnosis of AMI. To date, the detection of circulating cardiac troponins (such as troponin I and troponin T) is widely used for AMI diagnosis. However, troponin I is not a specific indicator of post-MI cardiac damage, since its plasma levels could eventually rise in chronic renal failure, pulmonary embolism and after non-cardiac surgery [59]. A high sensitivity troponin T assay is suggested to be a gold standard in early diagnosis of AMI due to the high sensitivity (on average, 89.5%) and high specificity (on average, 77.1%) at a cut-off value of 14 ng/L [60]. Plasma troponin could be detected as early as 4–8 h post-MI, reaching peak levels 18 h post-MI [61].

Cardiomyocytes produce many miRNAs; four of those (miR-1, miR-133a/b, miR-208a and miR-499) are specifically and/or abundantly expressed in these cells [62]. Cardiac-specific miRNAs are crucially involved in the regulation of cardiogenesis and heart function (*i.e.*, contractility and conductance) [63,64,65]. In cardiac injury, such as AMI, these miRNAs rapidly appear in the blood, indicating myocardial damage [66,67]. In AMI, plasma levels of cardiac-specific miRNAs are dramatically increased. For example, in AMI patients, plasma concentrations of miR-208b could be elevated by 1600-fold [66] or even by 3000-fold [68] compared to healthy individuals. In AMI, cardiac-specific miRNAs could be detected in blood earlier (<4 h post-MI) than cardiac troponins, indicating their potential advantage for the earliest diagnosis of this pathology [69]. In addition, several studies showed that detection of circulating cardiac-specific miRNAs in AMI has a better sensitivity and specificity than troponin T [70,71,72]. Furthermore, exosomal cardiac-specific miRNAs could be detected in AMI urine, while troponins are not, because these blood protein biomarkers are difficult to filter into urine [73]. Indeed, these observations suggest a potential value of circulating cardiac-specific miRNAs as promising diagnostic biomarkers for early diagnosis of AMI [74].

**Figure 1:**
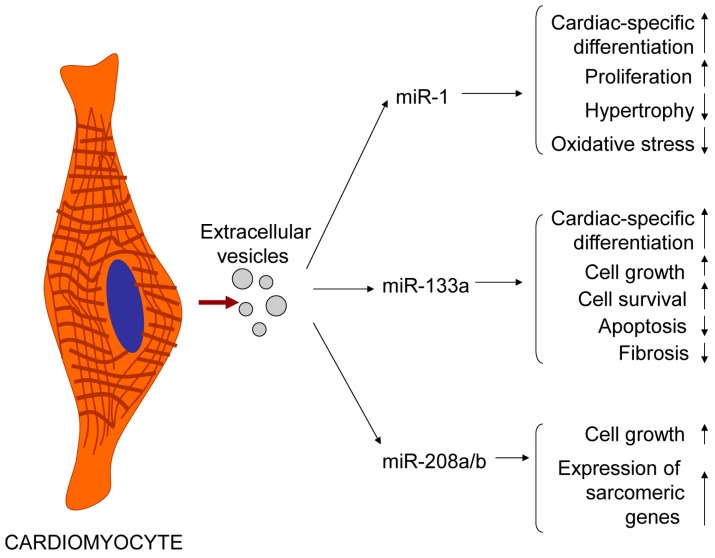
Cardioprotective properties of exosomal cardiac-specific microRNAs released by injured cardiomyocytes in acute myocardial infarction on cardiac muscle cells and resident cardiac stem/progenitor cells.

Exosomes released by injured cardiomyocytes are enriched with cardiac-specific miRNAs [67,73]. Increased EV released by damaged cardiac muscle cells reflects an adaptive response, warning other cells about heart injury. Cardiac-specific miRNAs regulate the expression of sarcomeric genes (such as miR-208 and miR-499) and ion channel genes involved in cardiac conductance, rhythmicity and automaticity (such as miR-1 and miR-133a). Cardiac-specific miRNAs also exhibit anti-apoptotic (miR-133a and miR-499), anti-fibrotic (miR-133a) and antioxidant (miR-1) properties (Figure 1) [75,76,77,78,79,80,81,82]. Thus, post-MI release of cardiac-specific miRNA-containing exosomes is essential for cardioprotection and induction of cardiac tissue repair.

Cardiac exosomes could serve as a valuable source of cardiac-specific miRNAs and other cardiac-specific markers for diagnostic purposes. EVs provide an extreme stability to their cargo components and indeed could be stored for a long time without visible degradation of the content [83].

However, it should be mentioned that circulating miRNAs may exist independently of exosomes. In the blood stream, miRNAs can be stabilized and protected from plasmatic ribonuclease through the assembly with high density lipoprotein (HDL) particles [84] or with at least two RNA-binding proteins, such as nucleophosmin 1 and AGO2 [85]. Indeed, along with exosome-associated miRNAs, the existence of exosome-free miRNAs could also suggest a contribution of extracellular miRNAs to communications between cells. This could be supported by the results of experiments showing functional activity of HDL- and Ago2-associated miRNAs in recipient cells [84,86].

## 10. Exosomes in Cardiac Remodelling/Fibrosis

Remodelling of injured myocardial tissue is an essential step in post-infarct heart healing. This process requires an intensive communication between cardiac fibroblasts, myofibroblasts, cardiac myocytes, ECs, immune cells and other cell types to control the process of clearance of the necrotic tissue and its replacement with the connective tissue. In fact, scar formation is a common mechanism of post-injury body repair. Other lineage cells can be mobilized in order to repopulate the lineage injured. In kidney injury, renal epithelial cells undergo epithelial-to-mesenchymal transition and initiate conversion of surrounding fibroblasts to myofibroblasts through promoting expression of type I collagen, F-actin and α-smooth muscle actin [87].

Similarly, cardiac ECs are subjected to EMT controlled by Snail, a transcription factor induced in the endothelium upon ischemia/reperfusion [57]. ECs also upregulate the secretion of connective tissue growth factor (CTGF) that activates the transformation of fibroblasts to myofibroblasts. In the infarcted heart, CTGF production is known to be increased, suggesting the role of this growth factor in cardiac fibrosis. TGF-β secreted by fibroblasts and cardiomyocytes stimulates CTGF expression [88]. As messengers for delivery of large “prosurvival” info-packages, exosomes are critically involved in tissue remodelling/fibrosis. TGF-β-containing exosomes were shown to activate the differentiation of fibroblasts to myofibroblasts [89] and profibrotic response in fibroblasts [90]. Cardiac EVs could also contribute to cardiac remodelling. As mentioned above, TGF-β-treated cardiomyocytes release exosomes enriched with RNA transcripts related to TGF-β-mediated signaling pathway [36]. After AMI, injured cardiac muscle cells secrete high levels of exosomes containing miR-208a that exhibits profibrotic effects in the heart [91]. However, TGF-β-dependent signaling can frequently lead to pathologic hypertrophy and even fibrosis. Indeed, post-MI cardiac remodelling should be strictly coordinated to avoid extensive irreversible structural changes in the myocardium. For example, cardiomyocytes release cardioprotective exosomes with a high content of miR-133a that suppresses cardiac fibrosis by targeting CTGF and type IA1 collagen [77]. In diabetic rats, Chaturvedi *et al.* [92] observed that exercise induces the secretion of cardiac exosomes containing anti-fibrotic miRNAs (miR-29b, miR-323-5p, miR-455 and miR-466). These miRNAs downregulate matrix metalloproteinase (MMP)-9 (an enzyme involved in extracellular matrix remodelling), whose production is elevated in diabetic heart.

In infarcted heart, a proper control of cardiac remodelling is frequently missing. Due to increased cardiac workload, this maladaptation could lead to the induction of arrhythmias, tachycardia, left ventricular hypertrophy and increased risk of heart failure [93]. In order to stimulate heart healing, cardiac fibroblasts and myocytes are shown to shed exosomes containing miRNAs (such as miR-21*, miR-208a and miR-499) with hypertrophic function [49,94]. However, due to the lack of a proper control, overproduction of these exosomes will favour the development of cardiac hypertrophy.

## 11. Exosomes in Cardiac Repair and Cardioprotection

In mammals, the adult heart contains resident cardiac stem cells (CSCs) and CPCs that could be involved in the repair of injured myocardium and contribute to replacing cardiomyocytes during aging. CSCs are positive for stem cell and progenitor markers, such as stem cell growth factor (SCF, also known as c-kit), stem cell antigen-1 (Sca-1), insulin gene enhancer protein (ISL1), *etc.* In suspension culture, these cells spontaneously form multi-cellular clusters (dubbed cardiospheres) and have the potential to differentiate into cardiac muscle cells, smooth muscle cells and endothelial cells [95]. Cultures of c-kit-positive CSCs isolated from the adult rat heart were able to maintain their stemness through multiple passages. Some, over long-term CSC cultures, upregulated the expression of cardiac lineage-specific transcription factor GATA-4 and differentiated into cardiomyocytes [96]. Indeed, the high regenerative capacity of CSCs and CPCs suggests their likely involvement in post-MI heart repair.

Rat CSCs cocultured with adult cardiomyocytes were shown to possess cardioprotective properties by increasing cardiomyocyte survival and inhibiting apoptosis through the release of various growth factors, including IGF-1, TGF-β and VEGF [96,97]. Exosomes released by CPC are likely to participate in the delivery of prosurvival and anti-apoptotic factors to cardiac muscle cells.

Similarly, Vicencio *et al.* [98] showed that plasma exosomes derived from healthy humans and adult rats are powerfully cardioprotective in all tested models of heart ischemia-reperfusion injury, since these vehicles are enriched with cardioprotective Hsp70. The mechanisms of plasma exosome-mediated cardioprotection involves Hsp70-dependent activation of Toll-like receptor 4 (TLR) followed by activation of cardioprotective Hsp27 in cardiomyocytes.

### 11.1. Cardioprotective and Regenerative Properties of Cardiac Progenitor Cell-Derived Exosomes

Electron microscopic observations showed that murine and human CPCs are able to produce exosome-like structures that are generated in MVBs [99,100]. These exosomes had a size of 30–90 nm and were enriched with proangiogenic miR-132, miR-146a and miR-210 that increased survival in cultured murine cardiomyocytes and induced tube formation in HUVEC cultures. Furthermore, treatment of rats with experimentally-induced IM by CPC-derived exosomes resulted in the improvement of cardiac function, less profound cardiac apoptosis and enhanced intracardiac angiogenesis [101]. miR-132 stimulates angiogenesis (especially in the presence of TGF-β, which induces miR-132 expression) via inhibition of p120RasGAP and derepression of the proangiogenic Ras-dependent mechanism [102,103,104]. The proangiogenic activity of miR-146a releases through targeting caspase recruitment domain-containing protein 10 (CARD10), an inhibitor of NF-κB that, in turn, impairs angiogenesis [105]. Hypoxia-induced miR-210 exhibits an anti-apoptotic effect by downregulating ephrin A3 [106] and caspase-8-associated protein 2 [107].

Gray *et al.* [108] observed that exosomes derived from CPCs cultured in hypoxic conditions had a more pronounced angiogenic potential than exosomes released by normoxic CPCs. Compared to normoxic exosomes, hypoxic exosomes contained higher amounts of proangiogenic miRNAs, including miR-132 and miR-146a, and could efficiently induce tube formation in cultured ECs and reduce the expression of profibrotic genes in fibroblasts. In a model of ischemia-reperfusion injury, treatment with hypoxic exosomes improved heart function and inhibited cardiac fibrosis [108]. Proangiogenic effects of CPC-derived exosomes on ECs resulted in the induction of the proliferative and migrative properties of ECs. Exosome-mediated migration of ECs is induced via activation of matrix metalloproteinases (MMPs) by exosomal CD147, an extracellular MMP inducer [40].

In rodent experimental models of MI, angiogenic, anti-apoptotic and anti-fibrotic properties of CPC-derived exosomes were also shown by other investigators [109,110]. The greatest regenerative efficiency of CPC-derived exosomes was observed when the exosomes were injected into the infarct border zone of mice at the time of MI. Overall, the exosomes improved heart function, decreased fibrotic area and increased the myocardial mass. Even injected when heart scarring is completed, CPC-derived exosomes still exhibited healing effects by reducing cardiac apoptosis and fibrosis.

Recently, Foglio *et al.* [111] observed that epicardiac progenitor cells also produce exosomes in response to IM-induced heart injury. In post-infarct, exosomes presented in the pericardial fluid exhibit cardioprotective effects by inducing epithelial-to-mesenchymal transition in epicardial cells, decreasing apoptosis and enhancing arteriogenesis. Epicardial exosomes were shown to contain clusterin, which plays a key role in TGF-β-dependent epithelial-to-mesenchymal transition [112] and anti-apoptosis [113].

Thus, during post-MI cardiac repair, resident progenitor cells release cardioprotective EVs in order to induce and promote myocardial regeneration. Since EVs influence various intracardiac cell types in a complex manner, these extracellular particles contain a variety of bioactive factors, most of which remain to be defined. Among these factors, exosomal miRNAs, such as miR-132, miR-146a, miR-451, miR-210, and others, play a prominent role in cardioprotection, post-MI neovascularization and heart healing.

### 11.2. Remote Ischemic Conditioning Enhances Cardioprotective Capacity of Exosomes

As mentioned above, exposure of CPCs to hypoxia induces the generation of exosomes with advanced cardioprotective properties [108]. Similarly, remote ischemic conditioning (RIC) (*i.e.*, repeated treating with brief episodes of ischemia/reperfusion) increases heart resistance to ischemic/hypoxic conditions. In rats, Yamaguchi *et al.* [114] used repeated RIC attempting to target MI-induced left ventricular remodelling. Repeated RIC was found to have a cardioprotective effect through decreasing cardiac remodelling and oxidative stress in infarcted hearts. Exosomes enriched with miR-29a (an anti-fibrotic miRNA) and IGF-1 receptor were involved in cardioprotection induced by repeated RIC [114]. miR-29a plays a key role in the negative regulation of fibrosis by targeting disintegrin metalloproteases ADAM12 and ADAM19 [115], TGF-β itself [116] and TGF-β-activated kinase 1-binding protein 1 (TAB1), which is an activator of the TGF-β-dependent profibrotic signaling [117].

Li *et al.* [118] found that RIC induces cardiac production of exosomes enriched with cardioprotective miR-144. Ischemia/reperfusion-induced injury downregulates myocardial miR-144 levels, but cardiac miR-144 content could be rescued by RIC. miR-144 was shown to increase cardioprotection through activation of PI3K/Akt and p44/p42 MAPK signaling, inhibition of the hypertrophic mammalian target of rapamycin (mTOR)-dependent pathway and stimulation of autophagy [119].

Pironti *et al.* [120] isolated exosomes from blood of mice subjected to heart pressure overload. These exosomes were enriched with angiotensin II type I receptor (AT1R), a crucial regulator of the cardiovascular function. AT1R-enriched exosomes were shown to be released from stressed heart and to serve AT1R delivery to cardiomyocytes and mesenteric resistance vessels for the induction of the adaptive response to pressure overload.

In summary, CPCs and cardiospheres containing CSCs and CPCs could serve as unlimited sources of cardioprotective exosomes. Exposure to hypoxia and stress enhances the cardioprotective potential of exosomes [121]. In experimental models of MI, intracardiac injection of CPC-derived exosomes showed beneficial effects by decreasing infarct size, pathological cardiac remodelling and apoptosis and promoting angiogenesis [101,108,110]. Indeed, these results suggest a promising therapeutic potential of cardiac exosomes for post-MI cardiac repair.

## 12. Conclusion: Perspectives of Using Cardiac and Non-Cardiac Exosomes in Cell-Free Cardiotherapy

The evaluation of the cardioprotective properties of exosomes in preclinical models of MI and other cardiac pathology has started recently. Many efforts should be made to provide evidence about the biosafety and efficiency of EV-based cardiotherapy in preclinical studies before moving to clinical trials. Studies of the bioactive molecular content of exosomes released by cardiac cells must be continued along with deciphering pathways and signaling mechanisms affected by these exosomes in acceptor cells. This will be helpful for a deeper understanding of the regenerative properties of cardiac exosomes.

Since exosomes from different cell sources carry major histocompatibility complex (MHC) molecules on their surface, some recipients could develop a low-grade immune reaction in response to exosome injection. In order to minimize the immunogenicity of exosomes, using allogeneic exosomes would be preferable. Furthermore, compared to cell transplants, exosome are poorly immunogenic, a feature that may provide an advantage for using these microparticles in cell-free regenerative therapy in a repeated dose manner [122].

In addition to CSCs and CPCs, non-cardiac stem cells, such as CD34-positive bone marrow cells [123] and mesenchymal stem cells (MSCs) [124], were used as a source for cardioprotective exosomes. Interestingly, exosomes from CD34-positive bone marrow cells were selectively taken up by cardiomyocytes and cardiac ECs, but not fibroblasts, suggesting the presence of cell-specific receptors on the exosomal membrane [123]. Indeed, these observations provide an option for targeted exosome-mediated delivery of cardioprotective drugs. In the case of MSCs, overexpression of the GATA-4 transgene was used to initiate predominant differentiation of these cells to the cardiac-specific lineage [124]. In summary, several stem/progenitor cell sources are available for the generation of cardioprotective exosomes. Further evaluations are required to select an optimal exosomal cell producer.

The clinical evaluation of exosomes in therapy is underway. At present, a number of exosome-based phase I/II clinical trials are being performed, especially in anti-cancer therapy. Exosomes shed by tumour cells (as a source of tumour-specific antigens) or exposure to a tumour-specific antigen is used to generate dendritic cells (DCs) that specifically recognize this antigen. DC-derived exosomes are isolated from conditioned media and then injected into cancer patients to induce an anti-tumour-specific immune response [125]. Overall, monotherapy or co-treatment with DC-derived exosomes showed good tolerogenicity and efficiency in the initiation of anti-tumour immunity [126,127].

At present, several promising strategies for the application of exosomes in cardiovascular therapy are under development. As mentioned above, stem-cell-derived exosomes have a great therapeutic potential for cardiac repair in regenerative medicine. Second, exosomes could be used as nanoparticles for targeted delivery of various bioactive molecules, such as miRNAs. For example, endothelial-specific miR-126 plays a key role in angiogenesis and vessel integrity and, therefore, could be used for vascular repair and regeneration [128]. Treatment of apolipoprotein E-deficient mice (an experimental model of atherosclerosis) with miR-126-loaded exosomes resulted in plaque stabilization, efficient incorporation of stem and progenitor cells into lesions and reendothelization [129,130]. Exosomes could be also used as carriers of therapeutically-important protein regulators, such as Sonic Hedgehog (Shh), a morphogen. This protein is crucially involved in the regulation of embryonic angiogenesis and cardioprotection and, in turn, might be helpful in promoting neovascularization in post-MI heart [131]. Proangiogenic, anti-apoptotic and vasculoprotective properties of Shh-containing EVs were demonstrated in murine models of experimentally-induced MI [132,133].

Generally, all of the above-mentioned approaches are equally important in the treatment of cardiovascular pathology. No doubt, lessons from clinical trials involving exosome-based strategies for anti-cancer therapy will be helpful for avoiding acute side effects of cardiovascular exosome-based therapy. Compared to cancer treatment, it is especially important to study exosome biodistribution in cardiovascular therapy, since the whole vascular network will be exposed to exosomes. The biosafety and tolerogenicity of exosome-based therapy is always crucial and needs to be strictly controlled in the translation from bench to bedside.
